# Ecological study measuring the association between conflict, environmental factors, and annual global cutaneous and mucocutaneous leishmaniasis incidence (2005–2022)

**DOI:** 10.1371/journal.pntd.0012549

**Published:** 2024-09-26

**Authors:** Maia C. Tarnas, Aula Abbara, Angel N. Desai, Daniel M. Parker

**Affiliations:** 1 Department of Population Health and Disease Prevention, University of California Irvine, California, United States of America; 2 Department of Infectious Diseases, Imperial College, London, United Kingdom; 3 Division of Infectious Diseases, University of California Davis Medical Center, Sacramento, California, United States of America; 4 Department of Epidemiology & Biostatistics, University of California Irvine, California, United States of America; Ohio State University, UNITED STATES OF AMERICA

## Abstract

**Background:**

Cutaneous and mucocutaneous leishmaniasis (CL/ML) cause significant morbidity globally and are vulnerable to changes from environmental events and conflict. In this ecological study, we aim to measure the associations between annual CL/ML cases, conflict intensity, and environmental factors between 2005 and 2022 globally.

**Methods:**

We pulled annual case data from the WHO for 52 nations that had conflict intensity scores (ranging from 1–10) from the Bertelsmann Transformation Index. Using Earth observation tools, we gathered temperature, precipitation, vegetation, and humidity data, in addition to data on annual estimates of population, internal displacement, and GDP. We fit a negative binomial generalized additive model with a random nation-level intercept.

**Results:**

Conflict was positively associated with increased CL/ML across the studied nations (IRR: 1.09, 95% CI: 1.01–1.16, p = 0.02). Given this, intense conflict (a score of ten) was associated with over double the risk of CL/ML compared to the lowest conflict levels (score of one). We also identified a curvilinear relationship between mean temperature and cases, as well as between vegetation level and cases. Each had small pockets of significant increased and decreased risk, respectively. Larger mean humidity ranges were negatively associated with cases. Importantly, the relationship between conflict intensity and cases was mediated by displacement.

**Discussion:**

Conflict is significantly associated with increased CL/ML cases. This is especially true at higher conflict levels, marking when conflict turns violent. The destruction of critical infrastructure (e.g., that related to healthcare, water, and sanitation) often seen during conflict could drive this association. Such environments can be hospitable to sandflies and can heighten individuals’ vulnerability through increased malnutrition, poverty, and displacement. Understanding this relationship is crucial for public health preparedness and response, especially as conflicts become increasingly violent and protracted.

## Author summary

Cases of cutaneous and mucocutaneous leishmaniasis, spread through sandflies infected with a *Leishmania* protozoan parasite, have risen in the past several decades. Though rarely fatal, cutaneous leishmaniasis causes highly stigmatizing skin lesions, and mucocutaneous leishmaniasis can destroy mucous membranes of the nose, mouth, and throat. Leishmaniasis is frequently found in areas with high levels of poverty, infrastructure destruction (e.g., of healthcare and water, sanitation, and hygiene facilities), displacement, and poor living conditions. Conflicts often create environments that are hospitable to leishmaniasis emergence, re-emergence, and transmission. Environmental factors, like temperature and precipitation, can also affect leishmaniasis incidence through impacts on the sandfly vector and on humans’ proximity to the vector. In our ecological study, we measured the association between conflict and leishmaniasis over 18 years while accounting for other factors, such as environmental conditions and population displacement, that are relevant to leishmaniasis’ spread. We found that conflict was significantly associated with increased risk of cutaneous and mucocutaneous leishmaniasis across the 52 included nations. Population displacement played an important role in the relationship between conflict and leishmaniasis incidence. This work is important for understanding global drivers of the increasing leishmaniasis burden in order to enhance preparedness efforts.

## Introduction

Leishmaniasis thrives in environments that conflict creates: those with poor living conditions, high malnutrition, and widespread poverty and displacement [1]. This vector-borne protozoal infection is spread by infected sandflies (namely of the *Phlebotomus* and *Lutzomyia* genera) and comes in three primary forms, of which cutaneous leishmaniasis (CL) is the most common [2]. CL causes lesions on the skin that easily scar and can lead to disability and stigma [3]. The World Health Organization (WHO) estimates that between 600,000 to 1 million new CL cases occur annually despite underreporting (only around 20–33% of cases are estimated to be reported), and are found primarily in the Eastern Mediterranean, Central Asia, and the Americas [3,4]. The second main form of leishmaniasis is mucocutaneous (ML), which can partially or completely destroy nose, mouth, and throat mucous membranes [3]. The majority of ML cases occur in Bolivia, Brazil, Ethiopia, and Peru [3]. Lastly, visceral leishmaniasis (VL) is the most severe form, with fever, severe splenomegaly and hepatomegaly, and anemia that can cause death in 95% of cases if left untreated. There is also significant underreporting of VL, with only 25–45% of the estimated 50,000–90,000 annual cases reported to WHO [3]. VL is thought to cause more deaths globally than any other neglected tropical disease [5]. Most cases occur in east Africa, India, and Brazil [3].

While the disease has existed throughout history, leishmaniasis cases have been rising globally in recent decades [6]. Conflict and environmental changes related to climate change are thought to be the primary contributors to this increase [1,3,7]. Higher global temperatures can widen the geographic range of the sandfly vector, leading to a larger distribution of leishmaniasis cases [6]. Temperature changes are also thought to increase parasite development in sandfly guts [8]. Deforestation and similar changes in land cover cause increased human interaction with vector habitats; the establishment of dwellings in or near areas of high vegetation has been associated with increased incidence of leishmaniasis [7,9]. This is because excess vegetation is conducive to sandfly breeding, as are precipitation and humidity (though too much precipitation can decrease vector populations) [9]. These environmental factors are all closely linked and often interact nonlinearly. Environmental phenomena also indirectly lead to increased leishmaniasis exposure via human population displacement because of droughts, floods, and famine; this disaster-related displacement can cause an influx of people into areas with high leishmaniasis transmission risk [3].

Vector-borne diseases generally do increase during conflict; following the onset of the Syrian conflict, reports of vector-borne diseases in Syria and neighboring nations rose by 458.5% [10]. Prior work by Berry and Berrang-Ford shows a significant dose-response relationship between leishmaniasis and levels of political terror and conflict [1]. This increase in leishmaniasis incidence, similar to increases in communicable disease incidence generally during conflict, results from a breakdown in water, sanitation, and hygiene (WASH), widespread infrastructure destruction (including that related to healthcare, WASH, and homes), displacement, poor living conditions, increased poverty, and interrupted vector control measures [1,11,12].

In this ecological study, we aim to explore the effects of conflict and environmental factors on CL and ML cases in nations reporting leishmaniasis cases to the WHO between 2005 and 2022. We focus on CL because of its global prevalence and presence in areas affected by conflict, though cases of ML are also included due to norms in case reporting. While prior studies have measured the associations between either environmental factors or conflict on leishmaniasis, none to our knowledge have analyzed both in tandem [1,13,14]. This study aids in understanding these joint effects on leishmaniasis, especially given that these factors will likely intertwine further in the future.

## Methods

This is a retrospective ecological study that uses routinely collected and reported public leishmaniasis data. We used a generalized additive model (GAM) with a negative binomial probability distribution and random nation-level intercept to model the relationship between conflict, environmental factors, and leishmaniasis cases at the nation level and across the globe while controlling for other relevant factors (also measured at the nation level). We included all nations that reported CL and ML cases to the WHO for at least half the years between 2005 and 2022 and for which conflict intensity scores were provided.

### Data sources

Nation-level CL and ML case counts between 2005–2022 (the outcome variable) were pulled from the WHO’s Global Health Observatory, which routinely collects annual counts of total reported cases from nations’ surveillance systems and Ministry of Health reports [15]. CL and ML case numbers are combined in WHO reporting and cannot be differentiated. Between 2005–2012, the WHO reported combined cases of imported and autochthonous leishmaniasis but separated imported cases beginning in 2013. To maintain consistency, we combined the imported and autochthonous cases for each nation from 2013 onwards; however, we did conduct a sensitivity analysis with just autochthonous cases between 2013–2022. The WHO reports cases from 90 total nations for CL and ML, but nations that had less than half of the reported years of cases or that were missing conflict intensity data were excluded from the analysis as their inclusion would have required us to drastically reduce the time series. Nation-years that were missing data were coded as missing and could be differentiated between years that had zero reported cases. Over the 18-year period, there were 107 nation-years (11%) with missing data. Case counts for Pakistan in 2017 were incomplete and we interpolated counts for that year by averaging cases from the preceding and succeeding years. We controlled for nations’ population size by using annual nation-level estimates from the World Bank, which were included in the model as an offset term [16].

In addition to the population offset, we included several covariates in this model (summarized in S1 Table). The Bertelsmann Transformation Index (BTI) reports a measure of nation-level conflict intensity every two years beginning in 2006 using insight from nearly 300 national and regional experts [17]. The conflict intensity score measures social, ethnic, and religious conflict severity within a nation on a scale of one to ten, where one indicates no violent social, ethnic, or religious-based incidents and ten indicates widespread violent conflict or civil war within the prior two years. BTI uses the following as indicators of conflict intensity: 1) “the confrontational nature of politics,” 2) “the polarization and split of society along one or several cleavages,” 3) “the mobilization of large groups of the population,” and 4) “the use and spread of violence” [18]. To have annual measurements, we averaged the conflict intensity scores for the years between reports; other tested interpolation techniques can be found in S2 Table. Conflict intensity was lagged one year based on scientific hypotheses and model fit (S3 Table) [1]. Briefly, this means that conflict in one year was used to potentially predict cases in the subsequent year.

To account for differences in nations’ wealth, we included each nation’s annual GDP, in US dollars, from the World Bank [19]. These were mean centered and standardized. We also included an estimate of the proportion of the total population internally displaced from conflict, violence, and natural disasters at the nation level from the Internal Displacement Monitoring Center [20]. We used the total number of internally displaced people at the end of each year to capture long-term displacement related to conflict and summed it with counts of post-disaster displacement to estimate total annual displacement. These data, which begin in 2008, are conservative estimates and likely underestimate the true amount of displacement seen within each nation. To account for scale differences related to varying population sizes between nations, we calculated the proportion of population displaced by dividing the total number of people displaced by total population for each year. These estimates were log-transformed to account for skewness.

Lastly, we utilized environmental data from several sources. Nation-level total observed precipitation and ambient temperature (mean, minimum, and maximum) were extracted from the World Bank’s Climate Change Knowledge Portal [21]. Minimum and maximum ambient temperature data were used to calculate a nation-level temperature range. We used NASA’s Global Land Data Assimilation System Land Surface Model to extract nation-level mean, minimum, and maximum measures of monthly specific humidity (originally collected at a 1°x1° spatial scale) in QGIS v.3.22.10 [22]. These monthly measures were transformed into annual mean humidity and humidity range. Lastly, we extracted normalized difference vegetation index (NDVI) data from NASA’s Terra MODIS satellite using Google Earth Engine [23]. The data were originally collected monthly at a 1km spatial scale, which we transformed into an annual nation-level measure. NDVI provides a measurement of vegetation levels on a scale of -1 to 1, where -1 indicates low surface vegetation and 1 indicates high surface vegetation. All measures were mean centered and standardized.

### Model building

To account for non-linear associations between variables and cases as well as the large number of zeros in our case data, we utilized a negative binomial GAM with CL and ML cases as the outcome, population (logged) as the offset term, and the remaining variables as model covariates. Since the analysis contains repeated observations within nations, we included a nation-level random intercept to account for likely differences in variance within and between nations. Initially, all variables were included as spline functions so as to not assume linearity. Variables that had a linear output were then put into the model as linear predictors to increase the model’s interpretability. The final model accounts for temporal, demographic, environmental, economic, and conflict-related measures. We also tested two interaction terms: one between conflict and displacement, and another between conflict and year. These interactions were not statistically significant and did not markedly improve model fit so were ultimately excluded (though each variable is included as a predictor). They can be found in S1 Fig. The model coefficients can be interpreted as incidence rate ratios (IRR). For continuous variables the coefficient indicates the IRR for a one-unit increase in the value for that variable. Analyses were done using R v.4.0.4.

## Results

### Descriptive epidemiology

**Fig 1:**
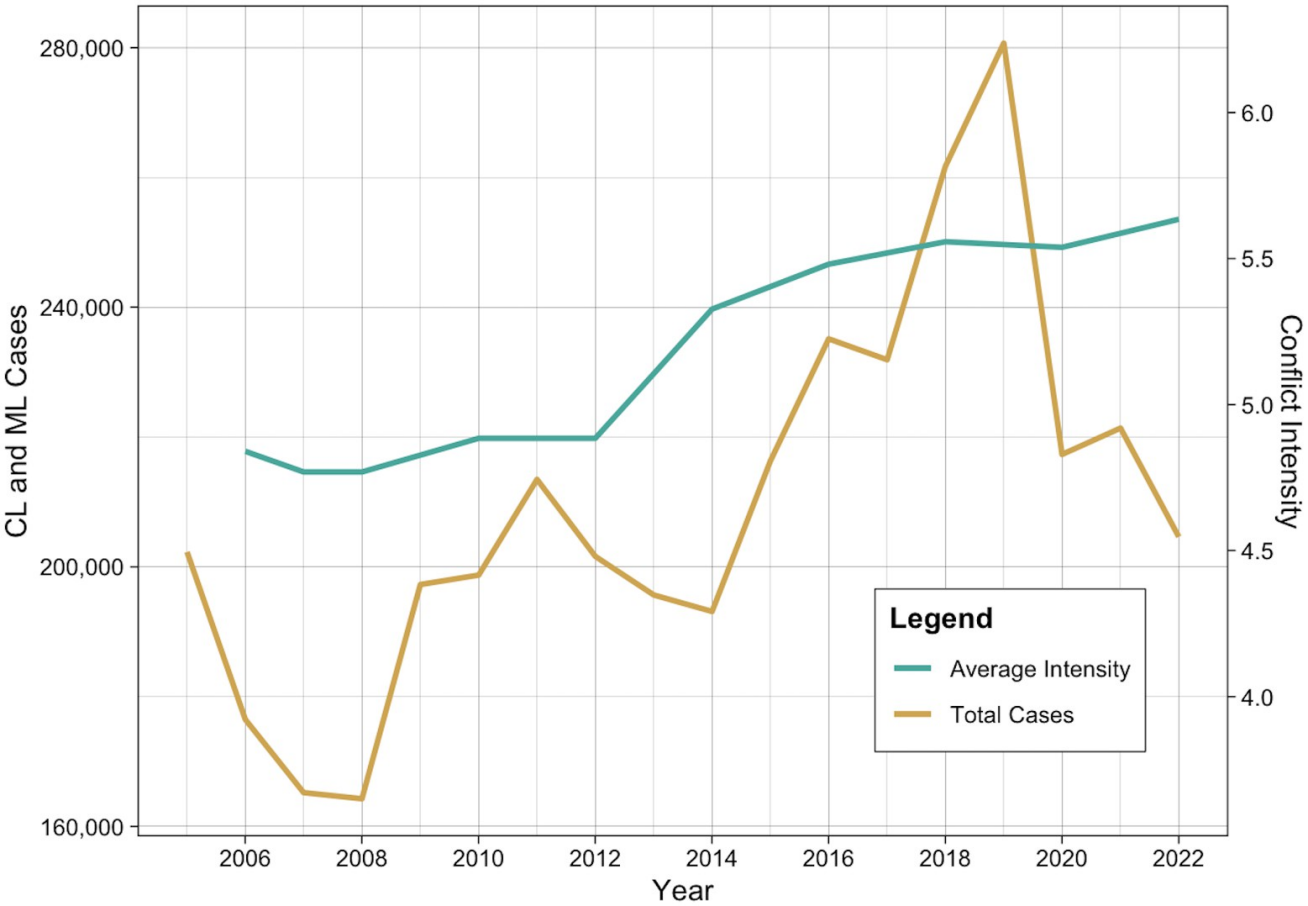
Leishmaniasis cases with average conflict intensity. Time series plot showing total number of cutaneous and mucocutaneous leishmaniasis cases over the study period with the average conflict intensity (not lagged) for each year.

Over the study period, the WHO reported 3,777,149 cases of CL and ML in the 52 nations included in the study (Fig 1). Only 21,963 cases (0.6%) were imported. The majority of the included nations were in Africa (n = 11) and the Middle East (n = 10), though the remainder of nations had a wide geographic spread between South America (n = 8), Central America (n = 7), Europe (n = 6), Central Asia (n = 5), South Asia (n = 4), and East Asia (n = 1). As of 2022, 51 of the 52 analyzed nations (98.1%, all but Ukraine) were classified as CL-endemic by the WHO [24].

**Fig 2:**
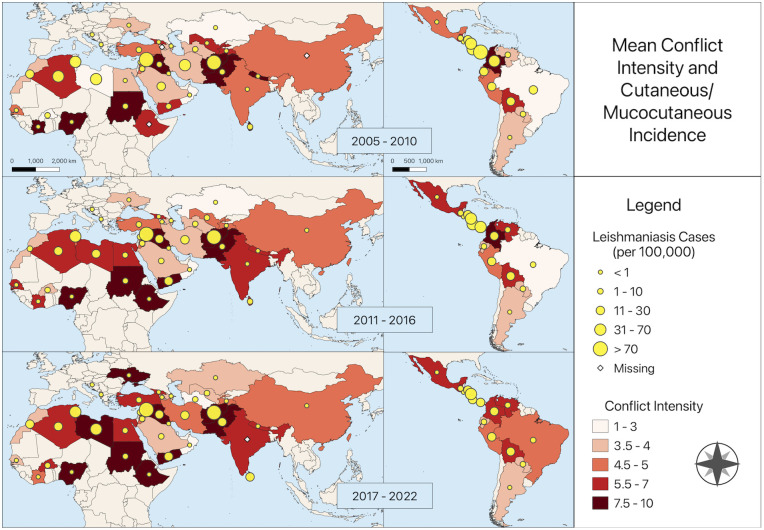
Map of mean conflict intensity and CL/ML incidence. Maps show incidence per 100,000 in 2005–2010, 2011–2016, and 2017–2022 for nations in the analysis. Maps were created using QGIS version 3.22.10. All layers were created by the authors.

Most cases were in Syria (n = 929,111; 24.6%), Afghanistan (n = 592,153; 15.7%), Brazil (n = 344,549; 9.1%), and Iran (n = 328,980; 8.7%). Syria also had the highest mean incidence (249.7 cases per 100,000), followed by Afghanistan (102.0 per 100,000), Nicaragua (52.4 per 100,000), and Tunisia (47.1 per 100,000) (Fig 2). Cases between 2020 and 2022 were relatively low, which may be due to the COVID-19 pandemic. Across all years, the mean conflict intensity score was 5.2 out of 10, though this increased over the study period (Fig 1). Mean conflict intensity was highest in Sudan (9.5), Afghanistan (9.2), Iraq (9.1), and Nigeria (8.4) and lowest in Oman (1.6) and Costa Rica (1.0) (Fig 2). Seven nations (Afghanistan, Côte d’Ivoire, Iraq, Libya, Sudan, Syria, and Yemen) reached the highest conflict intensity of 10 in at least one year.

### Model results

We sequentially added groups of variables to a base model, Model A, which contained year, conflict intensity, GDP, population (as an offset), and the random nation-level intercept (Table 1). This allowed us to assess model improvements and potential mediating effects between different groups of variables. In Model B, we added to Model A the proportion of population displaced, and in Model C subsequently added all the environmental variables (including 3 as splines). The model equations can be found in the Supporting Information (S1 Equations).

**Fig 3:**
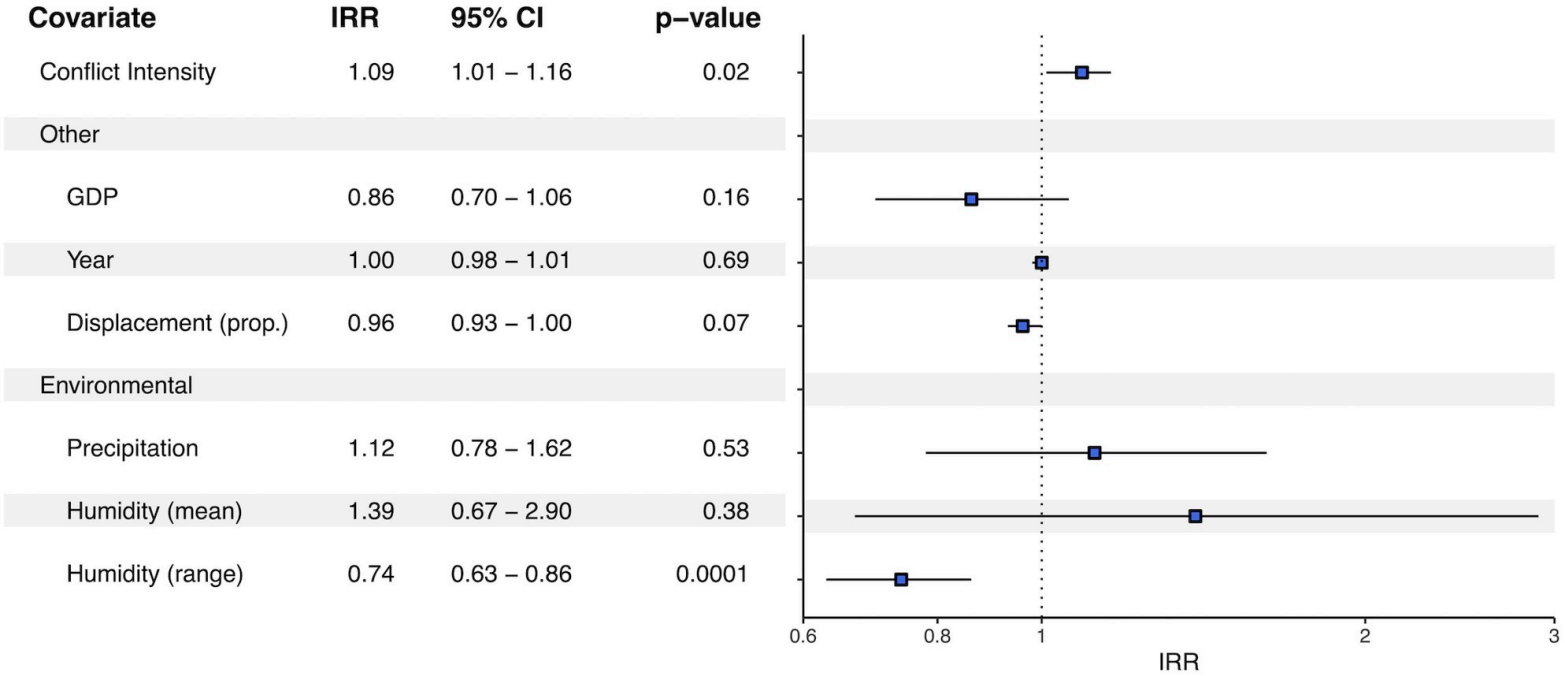
Forest plot of model output for cutaneous and mucocutaneous leishmaniasis. Plot shows the IRR and associated confidence intervals. Results are considered significant when the confidence interval does not go through the null of one. GDP = Gross Domestic Product. IRR = Incidence Rate Ratio.

Adding displacement to the model markedly improved overall model fit, as indicated by the lower Akaike’s Information Criterion (AIC) value in Model B versus Model A (Table 1). Results also remained consistent across each model. Conflict intensity was associated with increased CL/ML incidence in the following year. The conflict intensity levels are ranked from 1–10, and a one-unit increase in ranking was associated with a 9% increase in incidence (IRR: 1.09, 95% CI: 1.01–1.16, p = 0.03) (Fig 3). Nations with the most severe conflict levels (an intensity score of ten) had 2.09 (*e*^(0.0821^*^9)^) times greater risk of CL/ML than nations with a conflict intensity of one (very low conflict). When excluding data from 2020–2022 (the reporting of which was likely affected by COVID-19 [25]), this overall association increased slightly to 1.11 (95% CI: 1.02–1.20, p = 0.01, Table A in S1 Text), or a 2.54 (*e*^(0.1035^*^9)^) increase in risk between conflict intensities of one and ten. Similarly, this relationship held when only considering autochthonous cases between 2013–2022 (Table A in S2 Text). The interaction term between conflict and year—while not included in the final model—showed that the relationship between conflict and CL/ML cases has changed over time; in the beginning of the study period, conflict levels had to be higher to have a significant effect on cases, but in more recent years lower levels of conflict have had significant effects (S1 Fig).

**Table 1:** Results, including the IRR and 95% confidence intervals, for each linear variable throughout each model iteration (Models A, B, and C). AIC = Akaike Information Criterion, a model fit statistic where a lower number indicates better model fit.

Model	Model A		Model B		Model C	
Covariate	Year, conflict severity, GDP, population offset, and random nation-level intercept		Model A + displacement proportion		Model B + precipitation, temperature, humidity + NDVI	
	*IRR (95% CI)*	*p*	*IRR (95% CI)*	*p*	*IRR (95% CI)*	*p*
Conflict intensity	**1.06 (1.001–1.12)**	**0.0453**	**1.09 (1.02–1.17)**	**0.0119**	**1.09 (1.01–1.16)**	**0.0200**
GDP	0.85 (0.68–1.05)	0.1249	0.85 (0.69–1.05)	0.1335	0.86 (0.70–1.06)	0.1643
Year	1.00 (0.98–1.01)	0.6050	1.01 (0.99–1.02)	0.5345	1.00 (0.98–1.01)	0.6859
Displacement prop.			0.97 (0.93–1.01)	0.0976	0.96 (0.93–1.00)	0.0724
Precipitation					1.12 (0.78–1.62)	0.5318
Humidity (mean)					1.39 (0.67–2.90)	0.3771
Humidity (range)					**0.74 (0.63–0.86)**	**0.0001**
**AIC**	10,090.43		7,724.05		7,748.51	

**Fig 4:**
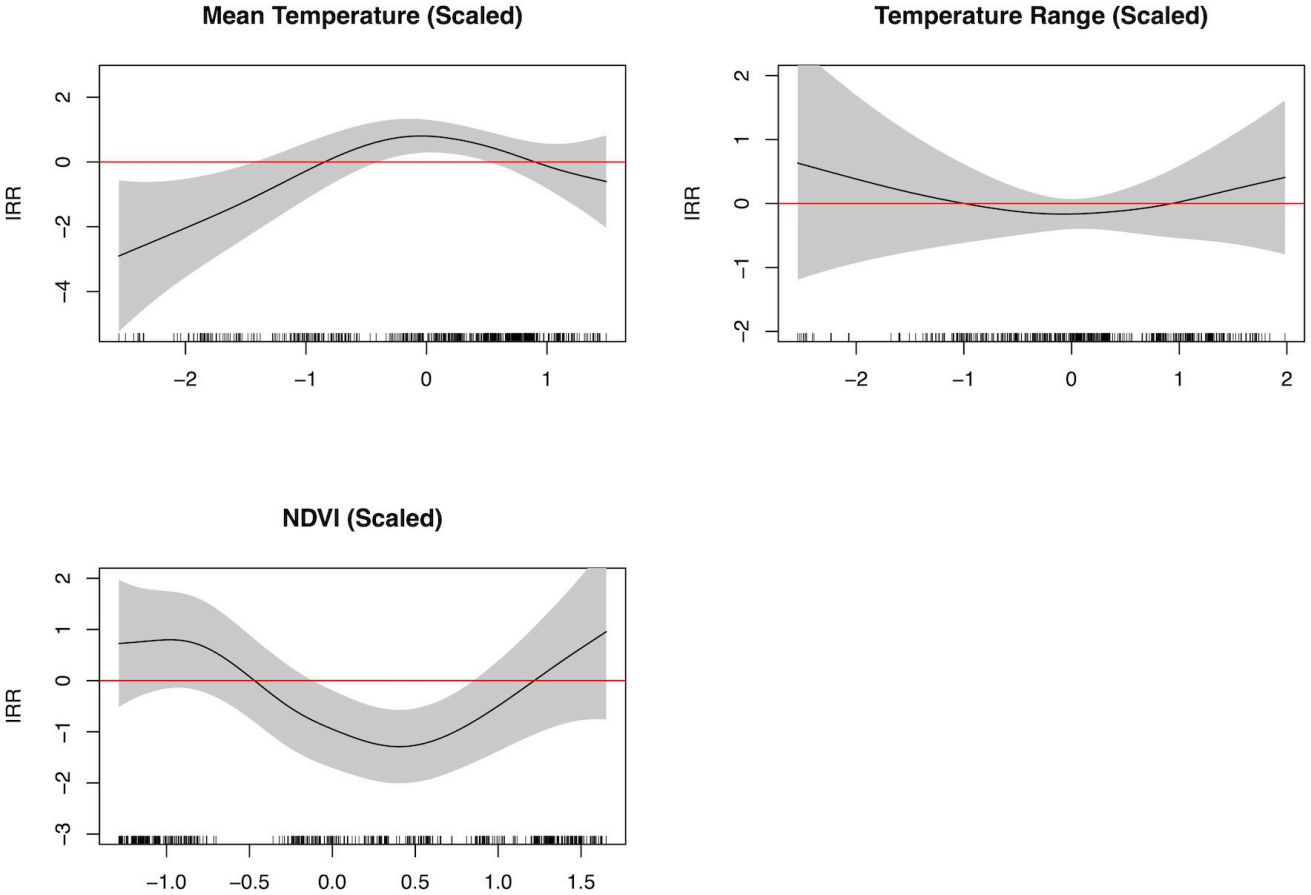
Spline outputs for the model. The variable is significant when both the black line and 95% confidence intervals (gray shaded area) are entirely above or below the red line.

In the spline outputs (Fig 4), lower mean temperature was largely protective against CL/ML cases. However, between approximately 17.02°C—23.58°C (-0.50 to 0.50 standard deviations from the mean), mean annual temperature was associated with increased risk of CL/ML cases. NDVI was negatively associated with CL/ML cases between levels of 0.34–0.57 (-0.25 to 0.75 standard deviations), or moderate vegetation levels.

## Discussion

Our results suggest that conflict is significantly associated with increased risk of CL/ML in the following year, even when accounting for relevant environmental factors, which is consistent with other literature [1]. This is especially true at conflict intensity’s highest levels, where conflict turns largely violent. The mechanisms for this are likely complex, though we hypothesize that conflict may create more appropriate breeding grounds for sandflies, cause wide-scale displacement (which we account for), and damage or destroy critical infrastructure. This includes healthcare and WASH-related infrastructure, which are important for preventing and appropriately treating leishmaniasis cases, and vector control infrastructure. We also found curvilinear associations between mean annual temperature and CL/ML, in addition to NDVI and CL/ML, which we hypothesize are related to optimal environments for sandfly vector populations [26,27]. As ectotherms, sandflies are sensitive to meteorological conditions, and the temperatures for which there was a positive association with CL/ML incidence (illustrated in Fig 4) align with the literature on optimal temperatures for sandfly survival and propagation [26,27]. Similarly, sandflies often prefer humid environments, and thus may thrive in environments with less variation in humidity levels as reflected in the model output [27].

In addition, conflict frequently increases poverty, which is strongly associated with CL [28]. The conflicts in both Yemen and Syria are good examples of this: following the onsets of their respective conflicts, 90% of the Syrian population has become impoverished, as has 80% of the Yemeni population [29,30]. Both conflict and poverty—independently and jointly—can exacerbate inadequate access to healthcare, WASH infrastructure, and sufficient housing, in addition to increasing forced displacement, coinfection with other infectious diseases (especially HIV), and malnutrition [28]. Vector control public health measures such as indoor residual spraying, breeding site reduction, and waste collection are often interrupted during conflict, which can create hospitable environments for sandflies while increasing individuals’ exposure to these environments. This is especially true in displaced communities. While displacement was not statistically significant in our model, it did act as an important mediator in the relationship between conflict and CL/ML incidence. Displacement increases with conflict intensity (S2 Fig), and thus conflict is likely indirectly affecting leishmaniasis incidence through displacement.

Conflicts globally are becoming more violent. Between 2006–2011, 23.2% of conflicts were a seven or above on BTI’s conflict intensity index, indicating when conflict turns largely violent. This rose to 28.9% between 2019–2024, reflecting a 24.2% increase (p < 0.05). This shift can also be seen in the percentage of conflicts that are at an intensity of three or below, indicating fairly low conflict levels: 28.4% of conflicts between 2006–2011 were at a conflict intensity of three or below, and this decreased to 19.2% and 12.8% in 2012–2018 and 2019–2024 respectively, showing that fewer conflicts remain small and non-violent. In 2024, 16 nations were involved in conflicts above an intensity level of seven; CL is endemic in each of these nations (curiously, CL is not considered endemic in Ukraine, despite a history of reported cases) [24]. Enhanced preparedness for leishmaniasis—and other infectious diseases—is therefore increasingly necessary as more conflicts rise to, and remain in, the highest severity category. This is also important to consider as climate change pushes more nations into the temperature range that enhances CL and ML risk [26].

Though CL is rarely fatal, it is a highly stigmatized disease [31–33]. As such, the necessity for prevention extends beyond the immediate clinical effects of the disease. Stigma from CL is associated with significant adverse mental health outcomes and decreased economic well-being [31,32]. Vicious cycles of poverty compound the marginalization of these communities from conflict. Women often face worse stigma from CL lesions and scarring than men, even frequently being considered unfit for marriage due to scarring [32]. This ostracization is of particular economic relevance, as women in several CL-endemic nations often have fewer economic opportunities than men and rely on marriage for social, economic, and familial stability.

### Strengths and limitations

Our study demonstrates an association between conflict intensity and reported CL/ML cases between 2005–2022; this builds on the minimal prior research that explores this association. However, there are several limitations. We relied on CL/ML reporting to WHO which, given challenges to diagnosis, healthcare access, and assessment in many of these settings, could reflect under- or over-reporting of cases. We were also unable to differentiate between cases of CL and ML in the WHO data. We used the BTI scale for conflict intensity which has some subjectivity and gives a nation-level score that does not reflect intra-nation variability. In the model, almost none of the environmental variables were statistically significant, but this may be due to their spatial and temporal scales; looking at the nation level may mask important environmental changes and events that occur at smaller spatial scales and impact local disease trends. This is also true of within-nation associations between the environment and conflict (i.e., in some nations, conflict parties may interact with the environment in ways that notably change them, such as through deforestation or troop movement). We encourage research that can investigate these relationships at smaller spatial scales.

We may also lack a sufficient time period over which to observe relevant environmental trends relating to climate change. However, the nation-level analysis offers an ecological view of the relationship between conflict, environmental events, and leishmaniasis that can serve as the basis of future research that operates at a smaller spatial scale. Though forced displacement did strongly modify our results when included in the model, we did not detect a significant effect despite it being frequently cited as a primary risk factor for CL and ML [1,12]. As with other variables, our nation-level unit of analysis and annual time frame may have been too large to capture the impact of displacement at smaller geographic or temporal scales.

## Conclusion

Conflict is significantly associated with increased incidence of cutaneous and mucocutaneous leishmaniasis globally, even when controlling for relevant economic, demographic, and environmental variables. Conflict is often marked by widespread violence and infrastructure destruction, which create hospitable environments for sandfly vectors and exacerbate individuals’ vulnerability through decreased access to healthcare and WASH infrastructure, in addition to increasing malnutrition, poverty, and displacement. As conflicts globally are becoming more violent and protracted [34–36], these findings have implications for future preparedness efforts. Our work reinforces the importance of adequate preparedness and response mechanisms for the prompt recognition of unexpected CL/ML increases. This is particularly so as the time between a sandfly bite and clinically overt infection may be prolonged. As such, by the time a clinical increase in cases is seen, the opportunity for effective prevention through vector control and public health measures may have been missed. Understanding the relationship between conflict and CL/ML trends can support public health preparation, particularly when climate change and other factors are considered.

## Supporting information

S1 Table Model variables. Description of variables included in the final model.
(PDF)

S2 Table Conflict intensity interpolation. BTI reports conflict intensity scores every two years beginning in 2006. We tested four different ways of interpolating the conflict intensity data between years in order to test the sensitivity of our model results to interpolation.
(PDF)

S3 Table Conflict lag specification. We experimented with two different lag specifications for the conflict variable in the model: no lag and lagged one year.
(PDF)

S1 Equations Equations for the final model. We sequentially added groups of variables to a base model (Model A) to assess model improvement and potential mediating effects between different groups of variables.
(PDF)

S1 Text Outputs for the final model run without COVID-19 years. As COVID-19 likely affected the reporting of leishmaniasis cases beginning in 2020, this date-restricted model assesses the studied relationships outside of this potential disruption.
(PDF)

S2 Text Outputs for the final model run without imported leishmaniasis cases. Between 2005–2012, WHO jointly reported cases of imported and autochthonous CL and ML. We ran the model from 2013–2022 with just the autochthonous cases.
(PDF)

S1 Fig Conflict intensity interaction terms. We tested two interaction terms: one between conflict intensity and year and the other between displacement and conflict intensity. Neither interaction term was included in the final model.
(PDF)

S2 Fig Conflict intensity and displacement. Scatterplots and boxplots showing the relationship between conflict intensity and displacement.
(PDF)
