# Supplementary material for: Ecological study measuring the association between conflict, environmental factors, and annual global cutaneous and mucocutaneous leishmaniasis incidence (2005–2022)
Source: PLoS Negl Trop Dis. 2024 Sep 26;18(9):e0012549. doi: 10.1371/journal.pntd.0012549 (PMC11460679; doi:10.1371/journal.pntd.0012549)
Supplement: S1 Table — Description of variables included in the final model. (PDF) [file pntd.0012549.s001.pdf]

**Model Variables**

| | Variable | Description | Source | Spatial Scale | Time |
|---|---|---|---|---|---|
| **Outcome** | case | Counts of reported cutaneous and mucocutaneous leishmaniasis cases (combined imported and autochthonous) | Cases: WHO Global Health Observatory [15] | Nation level | Annual |
| **Temporal** | year | Year, from 2005 to 2022 | | | Annual |
| **Economic** | gdp_scale | Annual gross domestic product in US dollars ($). Mean centered and standardized | World Bank [19] | Nation level | Annual |
| **Conflict Intensity** | con_intens | Conflict intensity rating ranging from 1 to 10 | Bertelsmann Transformation Index [17] | Nation level | Biennially; converted into annual measures by averaging scores in years between reports |
| **Environmental** *(all environ. variables are mean centered and standardized)* | precip | Total observed rainfall in mm | Climate Change Knowledge Portal (World Bank) [21] | Nation level | Annual |
| | temp_mean | Mean ambient temperature in °C | Climate Change Knowledge Portal (World Bank) [21] | Nation level | Annual |
| | temp_range | Range of ambient temperature in °C, calculated using minimum and maximum ambient temperature | Climate Change Knowledge Portal (World Bank) [21] | Nation level | Annual |
| | hum_mean | Mean specific humidity in g/kg | NASA's Global Land Data Assimilation System Land Surface Model [22] | 1° x 1°, mean taken at nation level | Monthly; converted to annual by averaging |
| | hum_range | Range of specific humidity in g/kg, calculated using minimum and maximum humidity | NASA's Global Land Data Assimilation System Land Surface Model [22] | 1° x 1°, range taken at nation level | Monthly; converted to annual by averaging |
| | ndvi | Normalized difference vegetation index, range from -1 to 1 | Terra MODIS satellite [23] | 1km, mean taken at nation level | Monthly; converted to annual by averaging |
| **Demographic** *(both are log-transformed)* | displace_prop | Proportion of the total population internally displaced at the end of each year. This includes conflict- and disaster-related displacement | Displacement: Internal Displacement Monitoring Center [20] Population: World Bank [16] | Nation level | Annual |
| | pop | Annual population estimate | World Bank [16] | Nation level | Annual |
| **Random intercept** | nation | | | Nation level | Constant |

**S1 Table:** Description of variables included in the final model
