# Supplementary material for: Ecological study measuring the association between conflict, environmental factors, and annual global cutaneous and mucocutaneous leishmaniasis incidence (2005–2022)
Source: PLoS Negl Trop Dis. 2024 Sep 26;18(9):e0012549. doi: 10.1371/journal.pntd.0012549 (PMC11460679; doi:10.1371/journal.pntd.0012549)
Supplement: S2 Table — BTI reports conflict intensity scores every two years beginning in 2006. We tested four different ways of interpolating the conflict intensity data between years in order to test the sensitivity of our model results to interpolation. (PDF) [file pntd.0012549.s002.pdf]

Model Specification: Conflict Intensity Interpolation

BTI reports conflict intensity scores every two years (on the even years), beginning in 2006. We tested four different ways of interpolating these data between years in order to test the sensitivity of our model results to interpolation. These methods included: 1) averaging conflict intensity scores between years, 2) applying the score from the prior even year to the following odd year ("forward score application"), 3) applying the score from the following even year to the prior odd year ("backward score application"), and 4) only using years for which conflict intensity scores were reported (i.e., the even years, with no interpolation at all). In each of these specifications, conflict intensity was lagged one year. Results for each specification are below (S2 Table). Note that each of these models also included splines for mean temperature, temperature range, and NDVI, though these are not shown for brevity. The model outputs for conflict intensity did not markedly change between tested specifications, and thus we utilized the first method in the final model.

**Covariate**	**Averaged Scores**		**Forward Score Application**		**Backward Score Application**		**No Interpolation**	
	IRR (95% CI)	*p*	*IRR (95% CI)*	*p*	*IRR (95% CI)*	*p*	*IRR (95% CI)*	*p*
Conflict intensity	**1.09 (1.01 – 1.16)**	**0.02**	**1.08 (1.01 – 1.15)**	**0.02**	1.07 (1.00 – 1.14)	0.05	**1.12 (1.01 – 1.24)**	**0.03**
GDP	0.86 (0.70 – 1.06)	0.16	0.86 (0.70 – 1.06)	0.16	0.86 (0.70 – 1.06)	0.16	0.80 (0.60 – 1.05)	0.11
Year	1.00 (0.98 – 1.01)	0.69	1.00 (0.98 – 1.01)	0.70	1.00 (0.98 – 1.02)	0.76	1.00 (0.97 – 1.03)	0.96
Displacement prop.	0.96 (0.93 – 1.00)	0.07	0.97 (0.93 – 1.01)	0.09	0.96 (0.93 – 1.01)	0.08	0.95 (0.88 – 1.02)	0.17
Precipitation	1.12 (0.78 – 1.62)	0.53	1.13 (0.78 – 1.63)	0.52	1.13 (0.78 – 1.63)	0.52	1.16 (0.63 – 2.14)	0.64
Humidity (mean)	1.39 (0.67 – 2.90)	0.38	1.41 (0.68 – 2.93)	0.36	1.39 (0.67 – 2.90)	0.38	1.22 (0.42 – 3.52)	0.72
Humidity (range)	**0.74 (0.63 – 0.86)**	**0.0001**	**0.74 (0.63 – 0.86)**	**0.0001**	**0.73 (0.63 – 0.86)**	**0.0001**	**0.69 (0.53 – 0.89)**	**0.004**

S2 Table: Outputs for each model specification.
