# Supplementary material for: Ecological study measuring the association between conflict, environmental factors, and annual global cutaneous and mucocutaneous leishmaniasis incidence (2005–2022)
Source: PLoS Negl Trop Dis. 2024 Sep 26;18(9):e0012549. doi: 10.1371/journal.pntd.0012549 (PMC11460679; doi:10.1371/journal.pntd.0012549)
Supplement: S3 Table — We experimented with two different lag specifications for the conflict variable in the model: no lag and lagged one year. (PDF) [file pntd.0012549.s003.pdf]

Model Specification: Conflict Lag

We experimented with two different lag specifications for the conflict variable in the model: no lag and lagged one year. We anticipated that lagging the conflict variable would be appropriate, since vectors would need time to reproduce in newly acquired niches and other hospitable environments following conflict-related destruction. However, we were interested in comparing the two model versions. As seen in S3 Table, the model with lagged conflict does result in a slightly better model fit. This also affirms that there is a delay between conflict and the associated increase in leishmaniasis incidence.

**Covariate**	**No Lag**		**Conflict Lagged 1 Year**	
	IRR (95% CI)	*p*	*IRR (95% CI)*	*p*
Conflict intensity	1.06 (0.99 – 1.14)	0.11	**1.09 (1.01 – 1.16)**	**0.02**
GDP	0.86 (0.70 – 1.06)	0.16	0.86 (0.70 – 1.06)	0.16
Year	1.00 (0.98 – 1.02)	0.84	1.00 (0.98 – 1.01)	0.69
Displacement prop.	0.97 (0.93 – 1.01)	0.10	0.96 (0.93 – 1.00)	0.07
Precipitation	1.14 (0.79 – 1.64)	0.50	1.12 (0.78 – 1.62)	0.53
Humidity (mean)	1.39 (0.67 – 2.91)	0.38	1.39 (0.67 – 2.90)	0.38
Humidity (range)	**0.73 (0.62 – 0.85)**	**< 0.0001**	**0.74 (0.63 – 0.86)**	**0.0001**
**AIC**	7,750.89		7,746.96	

S3 Table: Outputs for each model specification. The AIC (Akaike Information Criterion) indicates model fit, where a lower number signifies better fit.
