# Supplementary material for: Ecological study measuring the association between conflict, environmental factors, and annual global cutaneous and mucocutaneous leishmaniasis incidence (2005–2022)
Source: PLoS Negl Trop Dis. 2024 Sep 26;18(9):e0012549. doi: 10.1371/journal.pntd.0012549 (PMC11460679; doi:10.1371/journal.pntd.0012549)
Supplement: S1 Equations — We sequentially added groups of variables to a base model (Model A) to assess model improvement and potential mediating effects between different groups of variables. (PDF) [file pntd.0012549.s004.pdf]

Model Equations

Model A
cases of leishmaniasis = β_{0i} + β_1*(year)* + β_2*(gdp_scale)* + β_3*(con_intens)* + *log(pop)*

Model B
cases of leishmaniasis = β_{0i} + β_1*(year)* + β_2*(gdp_scale)* + β_3*(con_intens)* + β_4*(displace_prop_log)* + *log(pop)*

Model C (final model)
cases of leishmaniasis = β_{0i}+ β_1*(year)* + β_2*(gdp_scale)* + β_3*(con_intens)* +β_4*(displace_prop_log)* + β_5*(precip_scale)* + β_6*(hum_mean_scale)* + β_7*(hum_range_scale)* + f_1*(temp_mean_scale)* + f_2*(temp_range_scale)* + f_3*(ndvi_scale)* + *log(pop)*

Where β_{0i} is the random nation-level (i) intercept with a Gaussian distribution ($\beta_{0i} \sim N(\mu,\sigma^2)$); β_3 is lagged at t – 1; f_1 - f_3 are smooth functions estimated by the model using restricted maximum likelihood; and *log(pop)* is the offset term.
