# Supplementary material for: Ecological study measuring the association between conflict, environmental factors, and annual global cutaneous and mucocutaneous leishmaniasis incidence (2005–2022)
Source: PLoS Negl Trop Dis. 2024 Sep 26;18(9):e0012549. doi: 10.1371/journal.pntd.0012549 (PMC11460679; doi:10.1371/journal.pntd.0012549)
Supplement: S1 Text — As COVID-19 likely affected the reporting of leishmaniasis cases beginning in 2020, this date-restricted model assesses the studied relationships outside of this potential disruption. (PDF) [file pntd.0012549.s005.pdf]

Model from 2005 – 2019

COVID-19 likely affected the reporting of leishmaniasis cases between 2020 – 2022 (25). We ran our analysis with a restricted date range (2005 – 2019) to assess the relationships outside the influence of COVID-19 on case reporting.

**Covariate**	**No Lag**	
	IRR (95% CI)	*p*
Conflict intensity	**1.11 (1.02 – 1.20)**	**0.01**
GDP	**0.75 (0.58 – 0.97)**	**0.03**
Year	1.03 (1.00 – 1.06)	0.09
Displacement prop.	**0.95 (0.91 – 0.998)**	**0.04**
Precipitation	1.30 (0.87 – 1.94)	0.20
Humidity (mean)	1.40 (0.58 – 3.39)	0.45
Humidity (range)	**0.81 (0.66 – 1.01)**	**0.06**

Table A: Outputs for the date-restricted model

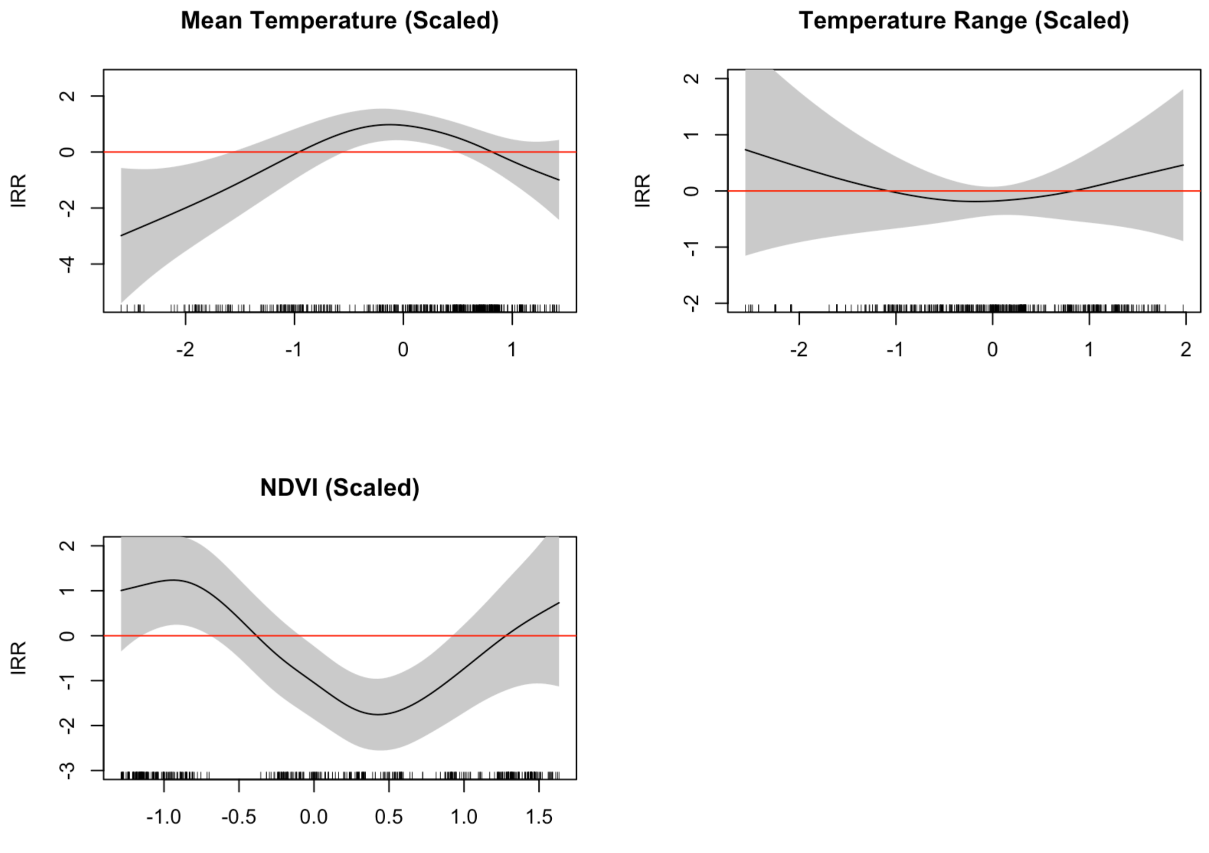

Figure A: Spline outputs from the date-restricted model for mean temperature, temperature range, and NDVI. The variable is significant when both the black line and 95% confidence intervals (gray shaded area) are entirely above or below the red line.
