# Supplementary material for: Ecological study measuring the association between conflict, environmental factors, and annual global cutaneous and mucocutaneous leishmaniasis incidence (2005–2022)
Source: PLoS Negl Trop Dis. 2024 Sep 26;18(9):e0012549. doi: 10.1371/journal.pntd.0012549 (PMC11460679; doi:10.1371/journal.pntd.0012549)
Supplement: S2 Text — Between 2005–2012, WHO jointly reported cases of imported and autochthonous CL and ML. We ran the model from 2013–2022 with just the autochthonous cases. (PDF) [file pntd.0012549.s006.pdf]

Model Without Imported Cases (2013 – 2022)

The WHO jointly reported imported and autochthonous CL and ML cases between 2005 and 2012 before separating them in 2013. We tested our model using just autochthonous cases between 2013 – 2022. Notably, when compared to the full model, GDP is negatively associated with CL/ML cases and humidity range is no longer significant.

**Covariate**	**No Lag**	
	IRR (95% CI)	*p*
Conflict intensity	**1.09 (1.01 – 1.19)**	**0.03**
GDP	**0.45 (0.25 – 0.82)**	**0.008**
Year	0.99 (0.97 – 1.02)	0.66
Displacement prop.	0.98 (0.93 – 1.02)	0.32
Precipitation	1.05 (0.70 – 1.56)	0.82
Humidity (mean)	1.00 (0.48 – 2.10)	0.9997
Humidity (range)	0.84 (0.66 – 1.07)	0.16

Table A: Outputs for the model without imported leishmaniasis cases

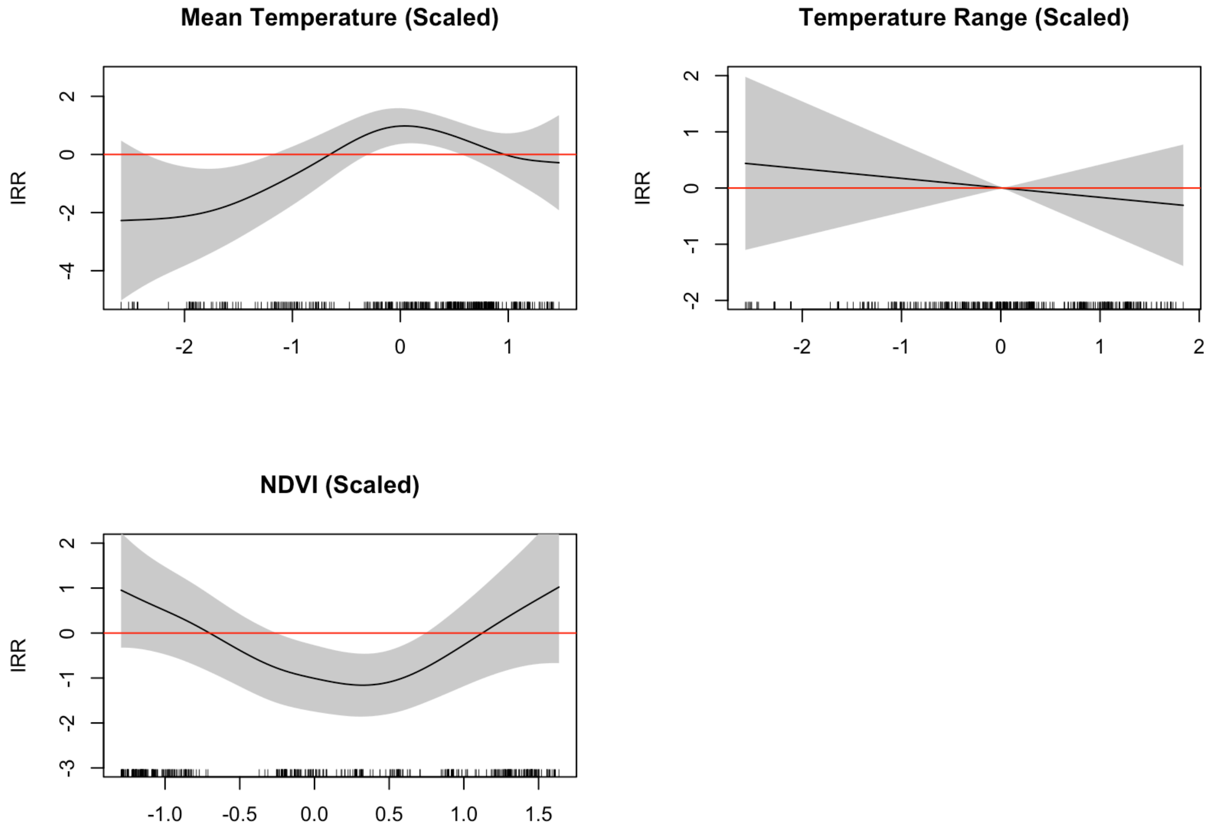

Figure A: Spline outputs from the model without imported leishmaniasis cases for mean temperature, temperature range, and NDVI. The variable is significant when both the black line and 95% confidence intervals (gray shaded area) are entirely above or below the red line.
