# Supplementary material for: Ecological study measuring the association between conflict, environmental factors, and annual global cutaneous and mucocutaneous leishmaniasis incidence (2005–2022)
Source: PLoS Negl Trop Dis. 2024 Sep 26;18(9):e0012549. doi: 10.1371/journal.pntd.0012549 (PMC11460679; doi:10.1371/journal.pntd.0012549)
Supplement: S1 Fig — We tested two interaction terms: one between conflict intensity and year and the other between displacement and conflict intensity. Neither interaction term was included in the final model. (PDF) [file pntd.0012549.s007.pdf]

Interaction Terms

During model specification, we tested two interaction terms: one between conflict intensity and year, and another between conflict intensity and displacement. Though we did not include either term in the final model based on model fit and interpretability, they both show interaction effects between the variables and the outcome (cases of leishmaniasis). The conflict and year interaction (S1 Figure) suggests that the relationship between conflict intensity and cases of leishmaniasis changed over the study period: in the beginning of the study period, conflict levels had to be higher to have a significant effect on cases, but in more recent years a lower level of conflict could have a significant effect. The conflict and displacement interaction (S1 Figure) indicates that when displacement is above a certain level and conflict is high, it has a positive association with leishmaniasis incidence. The highest levels of displacement (toward the right of the x-axis), however, do not show the strongest associations. Additionally, when conflict intensity is low, displacement has a negative association with leishmaniasis, even when displacement levels are high. This relationship is clearly complex, and factors such as reporting challenges and healthcare availability may play a role.

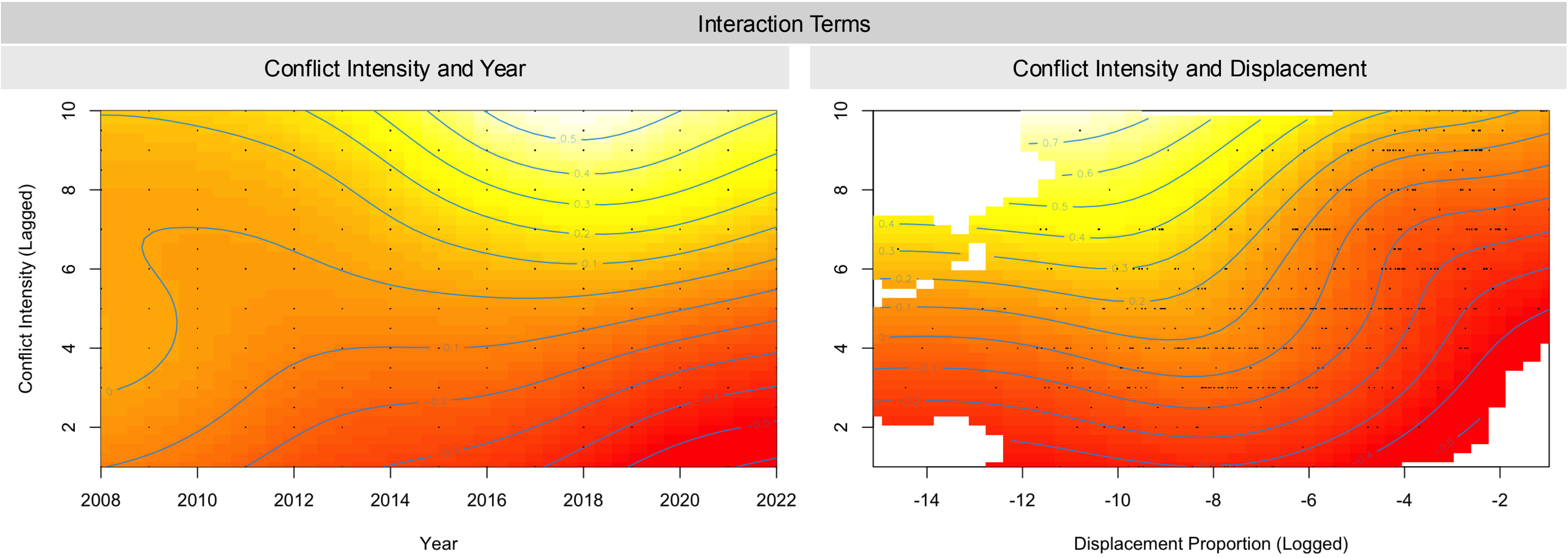

S1 Figure: Splines for the interactions between lagged conflict intensity and year (left) and lagged conflict intensity and displacement (right). Yellows indicate higher risk and reds indicate lower risk.
