# Supplementary material for: Ecological study measuring the association between conflict, environmental factors, and annual global cutaneous and mucocutaneous leishmaniasis incidence (2005–2022)
Source: PLoS Negl Trop Dis. 2024 Sep 26;18(9):e0012549. doi: 10.1371/journal.pntd.0012549 (PMC11460679; doi:10.1371/journal.pntd.0012549)
Supplement: S2 Fig — Scatterplots and boxplots showing the relationship between conflict intensity and displacement. (PDF) [file pntd.0012549.s008.pdf]

Displacement and Conflict

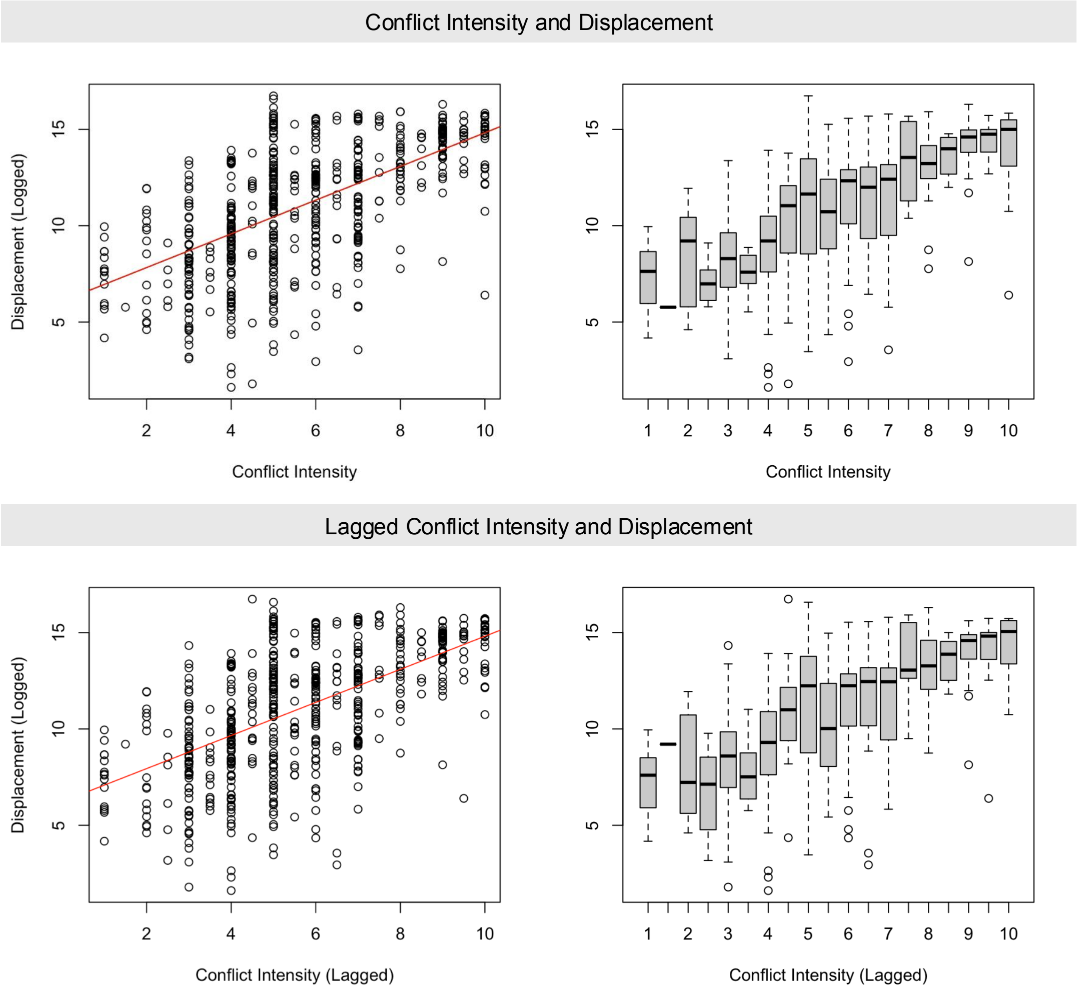

S2 Figure: Scatterplots with fitted regression lines and boxplots showing the relationship between conflict intensity and logged total displacement. The regression line slope when displacement and conflict intensity occur in the same year (top panel) is 0.8752 and is 0.8592 when conflict intensity is lagged one year (bottom panel).
